# Secondary Hemophagocytic Lymphohistiocytosis Complicated by Mucormycosis Following Liver Transplantation for Cryptogenic Acute Liver Failure: A Case Report

**DOI:** 10.1002/ccr3.73217

**Published:** 2026-07-27

**Authors:** Saif Ali Malik, Muhammad Hassaan Javaid, Syed Sibt E Haider, Hajirah Ghairat, AbuBakar Hafeez Bhatti, Hasibullah Aminpoor

**Affiliations:** ^1^ Department of Internal Medicine Shifa International Hospital Islamabad Pakistan; ^2^ Department of Medicine Shifa College of Medicine Islamabad Pakistan; ^3^ Department of Gastroenterology Shifa International Hospital Islamabad Pakistan; ^4^ Department of General Surgery Shifa International Hospital Islamabad Pakistan; ^5^ Department of Internal Medicine Rabia Balkhi National Complex Hospital Kabul Afghanistan; ^6^ Kabul University of Medical Sciences “Abu Ali Ibn Sina” Kabul Afghanistan

**Keywords:** cytomegalovirus, etoposide, hemophagocytic lymphohistiocytosis, HLH‐2004, H‐score, liver transplantation, mucormycosis, secondary HLH

## Abstract

HLH must be suspected in liver transplant patients who exhibit fever, cytopenia, hyperferritinemia, and dysfunction of the graft. HLH‐2004 guidelines and H‐score assessment early on will aid in early diagnosis and management, although prognosis remains poor when there are opportunistic infections.

## 1 Introduction

HLH is an acute, hyper‐inflammatory disease state that is caused by the uncontrolled activation of histiocytes and T cells, leading to a systemic “cytokine storm,” multisystemic dysfunction, and a high death rate [1]. HLH is characterized by nonspecific symptoms, such as fever, cytopenia, splenomegaly, and high ferritin levels [1]. The diagnosis of HLH is difficult in patients who underwent liver transplantation because fevers and cytopenias after liver transplantation have many differential diagnoses, and immunosuppression makes the classical picture of HLH difficult to detect [2]. In addition, secondary HLH is usually caused by infections with CMV or EBV, and sometimes MMF leads to HLH [3, 4, 5, 6].

The phenomenon of HLH following LT is still not well understood. Only 44 reported cases of post‐LT HLH were identified by Chesner et al. and Iseda et al., which include 24 adults and 20 children, of which only 5 patients showed signs of acute liver failure as their primary presentation [1, 2]. Considering the rarity of the condition, along with its diagnostic difficulties, high clinical suspicion and proper use of diagnostic scores will be necessary.

We describe the case of a 30‐year‐old woman who experienced HLH secondary to OLT for cryptogenic acute liver failure, which was complicated by fatal invasive mucormycosis. The current case demonstrates the difficulties in diagnosing posttransplant HLH, highlights the severe implications of immune suppression on patients, and stresses the importance of being alert to the potential development of invasive fungal infection during HLH treatment.

## 2 Case Presentation

A 30 year old female patient with a past medical history of bronchial asthma and no surgical history complained of progressive jaundice with bilateral pedal edema for 1 week. Clinical progressions can be seen in Table 1.

**TABLE 1:** Chronological timeline of clinical events.

Day/event	Clinical details
Presentation (Day 0)	30‐year‐old female with bronchial asthma; progressive jaundice and bilateral pedal edema ×1 week. Elevated transaminases, total bilirubin 37.90 mg/dL, INR 1.3.
Days 1–3 (Initial Workup)	Viral serology negative (HAV IgM, HBsAg, anti‐HCV, HSV IgM, EBV VCA IgM). ANA weakly positive; ASMA, anti‐LKM1, serum IgG, and liver biopsy excluded autoimmune hepatitis. Serum ceruloplasmin normal; no Kayser‐Fleischer rings—Wilson disease excluded. Acetaminophen undetectable. Metabolic work‐up (alpha‐1 antitrypsin, hereditary hemochromatosis) nondiagnostic. Abdominal ultrasound and MRCP: mild hepatomegaly, no biliary dilatation or focal lesion. Transjugular liver biopsy: ~50% hepatocyte necrosis consistent with acute liver injury. Diagnosis: cryptogenic acute liver failure (ALF).
Days 4–6 (transplant)	Clinical deterioration; INR 2.38 (worsening coagulopathy). Patient listed for orthotopic liver transplantation (OLT). OLT performed (8‐h procedure; estimated blood loss 3000 mL). Graft‐to‐recipient weight ratio: 1.00. Cold ischemia time: 62 min; warm ischemia time: 50 min. No immediate surgical complications. Immunosuppression initiated: ciclosporin + mycophenolate mofetil (MMF) + methylprednisolone. Post‐operative Doppler ultrasound: normal hepatic artery and portal vein flow; normal renal function.
Day 4 (post‐op)	Decreased Glasgow Coma Score necessitating intubation and mechanical ventilation.
Day 7 (HLH onset)	Fever > 38.5°C. Profound thrombocytopenia (platelets 8000/μL) and neutropenia (858/μL). AST 473 IU/L, ALT 992 IU/L. CMV PCR positive—intravenous ganciclovir commenced immediately. Cytopenias persisted despite antiviral therapy. Splenomegaly identified on repeat imaging. Bone marrow biopsy performed.
Days 8–10 (HLH Diagnosis)	Bone marrow biopsy: hemophagocytosis confirmed (H&E staining); CD68 immunostaining revealed marked histiocyte proliferation. Serum ferritin markedly elevated (2269 ng/mL). Triglycerides > 265 mg/dL. sIL‐2Rα significantly elevated. H‐score ≥ 228 (> 99% probability of reactive HLH). Five or more HLH‐2004 criteria met—secondary HLH diagnosed. EBV serology negative; no atypical lymphoid infiltrate on BM biopsy—PTLD excluded.
Days 10–11 (HLH Treatment)	HLH‐94 protocol initiated: intravenous dexamethasone (10 mg/m^2^/day) and etoposide (150 mg/m^2^ IV twice weekly). MMF dose reduced in view of myelosuppressive risk. Cytopenias worsened: leukocytes 150/μL, Hb 8.4 g/dL, platelets 47,000/μL.
Day 16 (mucormycosis)	Black discoloration of the surgical wound site noted. Histopathology of debrided tissue: broad, nonseptate, right‐angle‐branched hyphae on H&E staining—consistent with mucormycosis. Cultures confirmed Mucorales species. Galactomannan and beta‐D‐glucan testing nondiscriminatory for Mucorales. Liposomal amphotericin B commenced; surgical debridement performed.
Day 21 (death)	Progressive septic shock with multi‐organ failure. ICU admission with mechanical ventilation and inotropic support. Patient died on postoperative Day 21.

## 3 Methods

The differential diagnosis for cytopenias, fever, and hepatitis in the early posttransplantation period is vast. Using a systematic approach, the following potential diagnoses could be excluded prior to confirming a diagnosis of HLH. Acute cellular rejection (ACR) usually occurs within the first 2 weeks post‐OLT, with an increase in transaminase levels and jaundice, but without signs of haemophagocytosis, hyperferritinaemia (> 2000 ng/mL), hypertriglyceridaemia, or multilineage bone marrow depression. Haemophagocytosis and CD68 positivity detected by the bone marrow biopsy were inconsistent with the diagnosis of ACR. Since there was no percutaneous biopsy of the liver conducted to examine the portal tract for inflammation, eosinophilia, or damage to the biliary epithelium characteristic of ACR, this diagnosis could be ruled out at this point. Primary graft dysfunction (PGD) usually occurs within 72 h of transplantation and refers to a sudden onset of liver dysfunction due to ischemia and reperfusion injury. As proved by Doppler ultrasound, there were no abnormalities in hepatic artery and portal vein flow that would be consistent with such a diagnosis. Hematological decline on POD 7, outside of the period normally associated with PGD, along with haemophagocytosis observed from the bone marrow biopsy results, ruled out this diagnosis.

### 3.1 Drug‐Induced Cytopenias

Mycophenolate mofetil (MMF) is known to induce dose‐related bone marrow suppression, resulting in leucopenia and thrombocytopenia. Since MMF was part of the immunosuppression protocol and likely played a role in contributing to cytopenia levels, drug toxicity alone cannot account for the complete syndrome presentation, including haemophagocytosis, extreme elevation in ferritin levels (> 2000 ng/mL), hypertriglyceridaemia, and sIL‐2Rα. This drug was subsequently reduced in dosage once HLH was identified.

### 3.2 Cytopenias‐Related to Sepsis

While sepsis‐related immunosuppression may lead to cytopenias and raised serum ferritin levels, the severity of ferritinaemia (2269 ng/mL), the cytopenic profile (pancytopenia), the presence of haemophagocytosis on the bone marrow biopsy, and the raised levels of sIL‐2Rα are disproportionately high for the effects of sepsis alone. Mucormycosis, detected on POD 16, was believed to be a late‐onset complication rather than the primary cause of HLH. Posttransplant lymphoproliferative disorder (PTLD): Posttransplant lymphoproliferative disorder, especially that due to EBV, may have haemophagocytosis and associated cytopenia as part of its clinical presentation. In this case, the following findings exclude PTLD: negative EBV serology (VCA IgM); absence of atypical lymphoid infiltrate, signs of any hematological neoplasm, and organisms by Gram, PAS, and GMS stains on the bone marrow biopsy; and flow cytometry was not done but not clinically suspected either. The overall evidence supported a diagnosis of reactive (secondary) HLH rather than lymphoma‐associated HLH.

A dedicated HLH‐2004 diagnostic criteria summary table (Table 2) and H‐score calculation table (Table 3) are provided.

**TABLE 2:** HLH‐2004 diagnostic criteria: Patient assessment.

HLH‐2004 criterion	Threshold	Patient finding	Met?
1. Fever	Temperature > 38.5°C	Persistent fever ≥ 39.4°C post‐operatively	✓ Yes
2. Splenomegaly	Clinical or imaging evidence	Splenomegaly confirmed on repeat abdominal imaging (POD 7)	✓ Yes
3. Cytopenias (≥ 2 lineages)	Hb < 9 g/dL; platelets < 100 × 10^9^/L; neutrophils < 1.0 × 10^9^/L	Thrombocytopenia (8000/μL), neutropenia (858/μL), anemia (Hb not specified at POD7 but confirmed on BM biopsy day)	✓ Yes (3 lineages)
4. Hypertriglyceridaemia and/or hypofibrinogenaemia	Fasting triglycerides ≥ 3.0 mmol/L (> 265 mg/dL); fibrinogen ≤ 1.5 g/L	Triglycerides > 265 mg/dL (≥ 3.0 mmol/L). Note: Fibrinogen elevated (5.09 g/L), acute‐phase reactant effect in early/evolving HLH; criterion met via triglycerides.	✓ Yes (via hypertriglyceridaemia)
5. Haemophagocytosis on BM/spleen biopsy	Haemophagocytosis on histology	H&E staining of bone marrow biopsy confirmed macrophages engulfing erythrocytes and hematopoietic precursors. CD68 immunostaining: marked histiocyte proliferation.	✓ Yes
6. Low/absent NK‐cell activity	Below laboratory‐specific threshold	NK‐cell activity assay not available at the institution.	Not assessed
7. Hyperferritinaemia	Serum ferritin ≥ 500 ng/mL	Serum ferritin: 2269 ng/mL	✓ Yes
8. Elevated sIL‐2Rα (soluble CD25)	≥ 2400 U/mL	sIL‐2Rα significantly elevated (exact value not recorded)	✓ Yes
Total criteria met		7 of 8 criteria met (NK‐cell activity not assessed)	≥ 5 criteria: Diagnosis confirmed

**TABLE 3:** H‐score calculation.

Parameter	Possible values and points	Patient finding	Points assigned
1. Temperature (°C)	< 38.4 → 038.4–39.4 → 33 ≥ 39.4 → 49	≥ 39.4°C (persistent postoperative fever)	49
2. Organomegaly	None → 0Hepatomegaly or splenomegaly → 23Both → 38	Hepatomegaly on initial imaging; splenomegaly confirmed on repeat imaging (POD 7)	38
3. Cytopenias (≥ 2 cell lines)	1 lineage → 02 lineages → 243 lineages → 34	Thrombocytopenia (8000/μL), neutropenia (858/μL), anemia (confirmed at BM biopsy)	34
4. Ferritin (ng/mL)	< 2000 → 02,000–6000 → 35 ≥ 6000 → 50	2269 ng/mL → falls in 2000–6000 bracket	35
5. Triglycerides (mmol/L)	< 1.5 → 01.5–4.0 → 44 ≥ 4.0 → 64	> 265 mg/dL (> 3.0 mmol/L) → falls in 1.5–4.0 bracket	44
6. Fibrinogen (g/L)	≤ 2.5 → 30 > 2.5 → 0	5.09 g/L (acute‐phase reactant; see text)	0
7. AST (IU/L)	< 30 → 0 ≥ 30 → 19	473 IU/L (POD 7)	19
8. Haemophagocytosis on BM biopsy	No → 0Yes → 35	Confirmed (H&E, 40× magnification)	35
9. Known immunosuppression	No → 0Yes → 18	Post‐LT triple therapy (ciclosporin + MMF + methylprednisolone)	18
Total H‐score		H‐score ≥ 169 → > 93% probability of HLHH‐score ≥ 228 → > 99% probability	272

For fibrinogen, the patient exhibited hyperfibrinogenaemia (5.09 g/L; reference range 2.0–4.6 g/L), rather than the hypofibrinogenaemia specified in the HLH‐2004 criteria. This is a known occurrence in early/active HLH due to the higher rate of production of fibrinogen by the liver in response to the systemic inflammatory process than its rate of utilization by the activated macrophages. As described in the literature, fibrinogen is considered a systemic inflammatory acute phase reactant, and hypofibrinogenaemia will only be observed at a later stage of the condition. Fibrinogen criteria were thus not met. However, the hypertriglyceridaemia criteria (> 265 mg/dL) were independently fulfilled. Using the formula to calculate H‐score with the triglycerides and serum ferritin level (2269 ng/mL) obtained in this case, H‐score comes to 272 where ferritin belongs to the 2000–6000 ng/mL score range (35 points), not in the ≥ 6000 ng/mL range. The total H‐score is 272 (refer to Table 3), which again surpasses the cut‐off point for > 99% diagnostic probability.

Cellularity in bone marrow aspirate was increased; however, there were no signs of malignancy in any cells. No microbes were found in staining done by Gram, PAS, or GMS techniques. Flow cytometry was not performed. The diagnosis of EBV‐induced lymphoid proliferation was ruled out (as stated in Section 2.4).

Secondary HLH diagnosis was confirmed, and HLH‐94 therapy was started with 10 mg/m^2^/day of IV dexamethasone and twice‐weekly 150 mg/m^2^ of IV etoposide. Ciclosporin doses were titrated to protect the graft while minimizing additional immunosuppressive effects. Doses of MMF were also reduced. Concurrently, ganciclovir therapy via IV for CMV infection was continued.

Initial biochemical assessment showed marked elevation in hepatic transaminases, high serum bilirubin (37.90 mg/dL), and international normalized ratio (INR) of 1.3. An organized search for etiology was performed. Viral serologies were negative for hepatitis A virus (HAV IgM), hepatitis B surface antigen (HBsAg), anti‐hepatitis C antibody (anti‐HCV), herpes simplex virus (HSV IgM), and Epstein–Barr virus viral capsid antigen IgM (EBV VCA IgM). Testing for antinuclear antibody (ANA) was positive but weak; while ASMA, anti‐LKM1, serum IgG measurement, and liver biopsy ruled out autoimmune hepatitis. Wilson's disease was excluded by the presence of normal caeruloplasmin and lack of Kayser Fleischer rings. No measurable level of acetaminophen was found in the serum. There was no significant drug and toxins exposure. Evaluation for metabolic causes including alpha‐1 antitrypsin deficiency and hereditary hemochromatosis yielded negative results.

Abdominal ultrasound and MR cholangiopancreatography revealed mild hepatomegaly without biliary dilatation or focal hepatic lesions. Liver biopsy performed via transjugular approach showed 50% hepatocyte necrosis suggestive of acute liver damage of undetermined etiology. As a consequence of deteriorating patient status, ongoing coagulopathy (INR 2.38), and lack of spontaneous recovery of liver function, the patient was put on the waiting list for liver transplantation. The diagnosis of cryptogenic ALF was confirmed.

The patient was subjected to OLT surgery in an 8 h period. Intra‐operative bleeding during surgery was estimated at 3000 mL. The graft to recipient ratio was 1.00. Cold ischaemia time was 62 min while the warm ischaemia time was 50 min. There were no immediate complications from surgery. Due to decreasing GCS scores on POD 4, intubation was required, with mechanical ventilation instituted.

The treatment regimen with standard three‐drug immunosuppression, which included cyclosporine, mycophenolate mofetil, and methylprednisolone, was started. The Doppler ultrasonography examination conducted after surgery showed that the hepatic artery and portal vein flow were patent. Renal function was preserved.

It should be pointed out that information about the CMV status of both the donor and recipient as well as the CMV viral load at the time of the transplant, as well as details on any CMV prophylaxis regimen used (if any), were not available from the patient's medical records. This is a critical deficiency in this case that is analyzed in greater detail in Section 3. The hospital had a protocol in place regarding CMV prophylaxis during solid organ transplants using valganciclovir, especially when dealing with D+/R− pairs, but their application in this specific case could not be confirmed from available documentation.

Febrile illness (fever > 38.5°C) associated with marked thrombocytopenia (platelets 8000/μL) and neutropenia (neutrophils 858/μL) and with raised hepatic transaminases (AST 473 IU/L; ALT 992 IU/L) was recorded during POD 7. In the presence of the patient's immunocompromised status and the lack of explanation for the cytopenias, a test for CMV PCR was performed and the results were positive. Ganciclovir was administered intravenously. Imaging at this point also revealed splenomegaly, which was not present in the MRCP done before the transplantation procedure. Persistent cytopenias despite antiviral therapy, together with progressive biochemical deterioration, prompted bone marrow biopsy.

**FIGURE 1:**
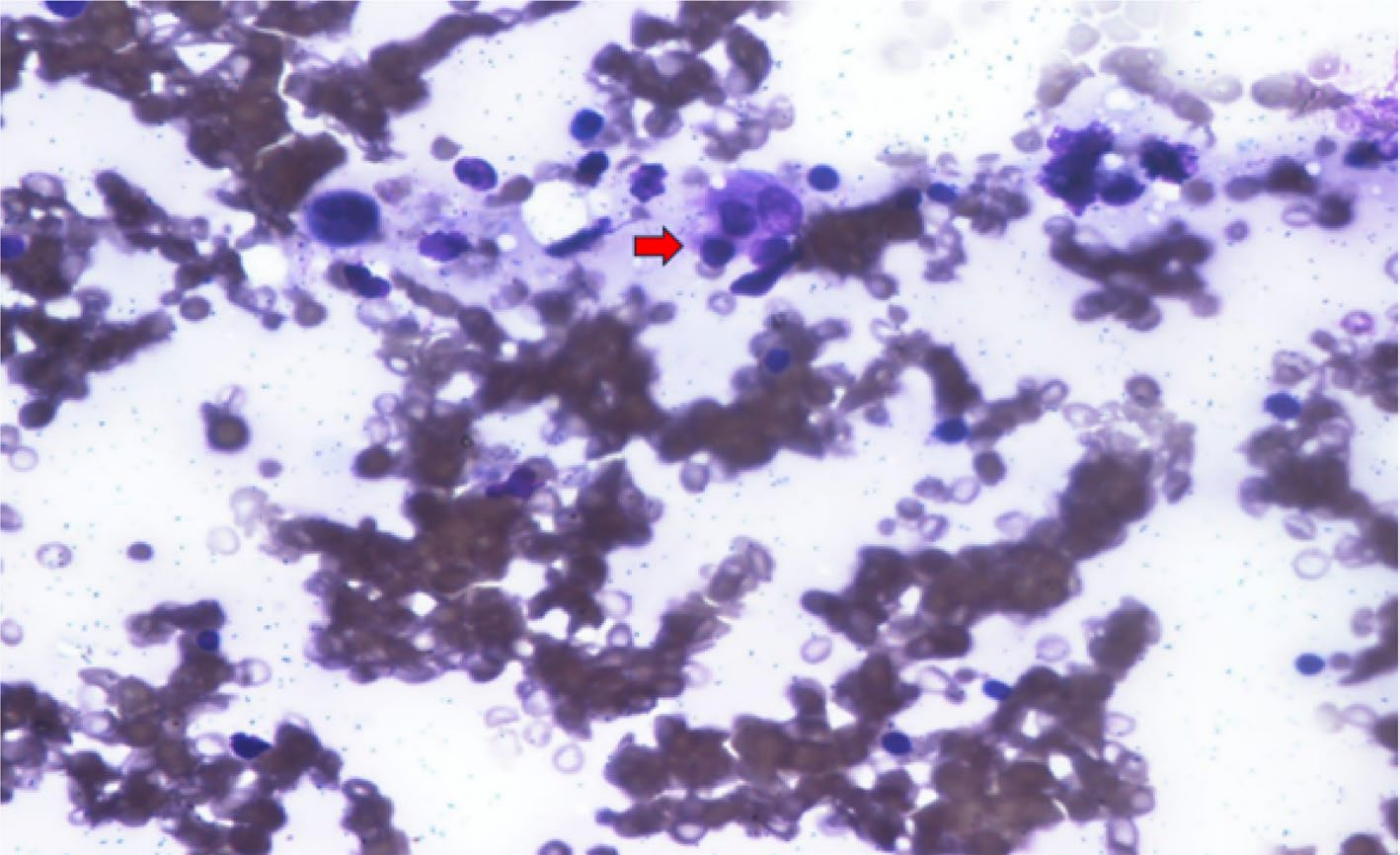
Haemophagocytosis in Bone Marrow Biopsy (H&E stain, 40× magnification). Hematoxylin and eosin staining demonstrates haemophagocytosis, the hallmark histopathological finding of HLH. Arrows indicate macrophages actively phagocytosing erythrocytes and other hematopoietic cells. Marrow cellularity was increased; no features of hematological malignancy were identified.

**FIGURE 2:**
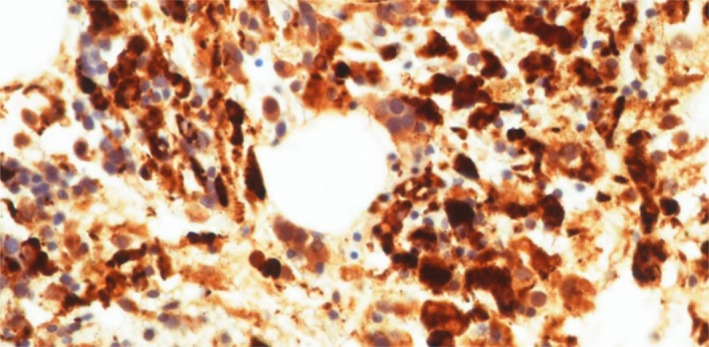
Proliferation of CD68‐Positive Histiocytes (Immunohistochemistry, 40× magnification). Immunohistochemical staining for CD68 highlights aberrantly proliferating histiocytes in the bone marrow biopsy specimen. Brown chromogenic staining identifies macrophages. The marked increase in CD68‐positive cells reflects the hyperinflammatory macrophage activation characteristic of HLH.

The H&E staining of the bone marrow biopsy sample showed definite hemophagocytosis by macrophages that had engulfed red blood cells and other hematopoietic cells (Figure 1). There was an increased proliferation of histiocytes detected using the CD68 immunohistochemical stain, which is characteristic of activated macrophages (Figure 2). The serum ferritin level was very high at 2269 ng/mL. Triglycerides were > 265 mg/dL. Soluble interleukin‐2 receptor alpha (sIL‐2Rα/soluble CD25) was significantly elevated.

Seven out of eight HLH‐2004 criteria were met, giving an H score of 272 (> 99% likelihood of secondary HLH) (see Tables 2 and 3). Diagnosis of HLH was established. Evaluation of NK cell function was not possible at the institution.

## 4 Results and Conclusions

Even with treatment, the cytopenias continued to deteriorate (WBC: 150/μL, hemoglobin level: 8.4 g/dL, platelet level: 47,000/μL), owing to the myelosuppression seen from both HLH and etoposide toxicity. Discoloration of the surgical incision site appeared by Day 16 of surgery, turning black. Histological study of the surgically removed specimen showed nonseptate, right‐angle branching hyphae on H&E stain, indicating invasive mucormycosis. The culture of the specimen also supported mucorales. Testing with galactomannan and beta‐D‐glucan did not aid in the diagnosis because the tests are not sensitive nor specific for mucorales infection. Liposomal amphotericin B was commenced and surgical debridement was performed.

Her condition progressed towards severe septic shock and multi‐organ dysfunction. She was intubated and placed in the ICU where she eventually succumbed to her disease process on POD 21. Laboratory findings on the day of her death were as follows (Table 4).

**TABLE 4:** Laboratory values on day of death (POD 21).

Parameter	Value	Unit	Reference range
Fibrinogen	5.09	g/L	2.0–4.6 g/L
Ferritin	2269	ng/mL	30–400 ng/mL
WBC count	0.10	×10^9^/L	4.0–10.5 × 10^9^/L
RBC count	2.47	×10^12^/L	3.8–5.8 × 10^12^/L
Hemoglobin	7.1	g/dL	12.5–16.0 g/dL
Hematocrit (HCT)	20.1	%	37%–47%
MCV	81.0	fL	78–100 fL
MCH	28.7	pg	27–31 pg
MCHC	35.5	g/dL	32–36 g/dL
Platelet count	6	×10^9^/L	150–400 × 10^9^/L

## 5 Discussion

HLH is one of the most difficult diagnoses to make in patients receiving liver transplants as it has overlapping clinical symptoms with graft dysfunction, acute cell‐mediated rejection, drug‐induced side effects, and infections [3]. Although infrequent, it is seen at a higher rate in solid organ transplant recipients (0.37%–1.72%) when compared to the overall population [7, 8]. To date, there have been 179 reported cases of posttransplant HLH, with kidney transplant recipients having the most (118 cases), while those receiving liver transplants comprise a lesser number [6, 7].

The higher risk is primarily due to the state of immunosuppression, making patients prone to opportunistic infections that act as major factors causing secondary HLH [4, 5]. Of these, the pathogens CMV and EBV are reported the most [9, 10]. The present case shows that the presence of concurrent CMV infection was the cause for the triggering factor as CMV has the potential to affect the functioning of NK cells as well as the activation of macrophages, causing hyperinflammation [11, 12, 13].

The use of immunosuppressive drugs could also play a role. Mycophenolate mofetil is a drug that suppresses the proliferation of T‐lymphocytes through inosine monophosphate dehydrogenase inhibition and has been linked to HLH post kidney transplantation [6, 14]. The same can happen due to the inability to regulate cytotoxic T‐cells and NK‐cells, which would lead to macrophages' activation [3, 15].

Regrettably, data about the donor and recipient's CMV status, viral load, and prophylaxis before and at the time of transplantation was not available. Nowadays, it is recommended that valganciclovir should be administered prophylactically for 3–6 months in high‐risk groups; that is, donors positive and recipients negative [10, 16, 17].

In relation to the diagnosis of the disease, the criteria of HLH‐2004, which expanded the HLH‐94 criteria from six to eight parameters, require meeting at least five criteria [15]. In our case, there were seven out of eight criteria fulfilled, while NK‐cell activity could not be estimated at that institution. According to the H‐score, an independently verified probabilistic approach, the patient scored 272, resulting in a diagnostic probability above 99% for reactive HLH [18]. It is important to mention that the H‐score calculation used the measured value of ferritin as 2269 ng/mL, with 35 points allocated as the parameter belongs to the range of 2000–6000 ng/mL. Moreover, the level of triglycerides was higher than 265 mg/dL and, therefore, scored as 1.5–4.0 mmol/L (44 points). In the previous calculation of H‐score, the ferritin level in the range of ≥ 6000 ng/mL was wrongly placed. According to the measured serum ferritin level of 2269 ng/mL, the right categorization would be 2000–6000 ng/mL, having a score of 35 points. The new calculation produced an H‐score of 272, which still exceeded the required cut‐off for HLH diagnosis.

However, the fact that there was hyperfibrinogenaemia (5.09 g/L) instead of hypofibrinogenaemia calls for special mention. Fibrinogen is an acute‐phase protein produced by the liver, and in the early or developing phase of HLH, especially the transplant type, where there is high systemic inflammation load, fibrinogen production can exceed its consumption by the activated macrophages. Hence, normal or increased levels of fibrinogen have been noted in HLH and do not exclude its diagnosis if other criteria are satisfied [19, 20, 21]. The results of each individual test must be considered within the context of the overall diagnostic process.

The occurrence of mucormycosis on POD 16 serves as an illustrative example of immunological catastrophe in this case report. The following three factors independently combined to cause severe immunosuppression: (i) use of calcineurin inhibitors and antimetabolites for prevention of graft rejection; (ii) immune paralysis due to HLH‐mediated suppression of NK cells and T cells, in addition to hypercytokinaemia; and (iii) etoposide‐induced myelosuppression. Mucormycosis is classically associated with infection with Rhizopus and other Mucorales, which can be fatal in over 50% of cases among SOT recipients [22, 23, 24]. Presentation of black eschar must prompt immediate histopathology, treatment with lipid formulation of amphotericin B, and surgical debridement. Tests for galactomannan and beta‐D‐glucan lack reliability for Mucorales and therefore cannot exclude this condition alone [23].

In liver transplant recipients, HLH treatment is challenging because immunosuppression is required to prevent graft rejection, while immunomodulation is needed to control the cytokine storm. The standard HLH‐94/HLH‐2004 regimen includes dexamethasone, etoposide, and selected use of ciclosporin, with an estimated 5‐year survival of 61% [15, 25]. However, etoposide is hepatotoxic and markedly myelosuppressive, limiting its use after transplantation. Emerging evidence supports targeted therapies such as the JAK1/2 inhibitor ruxolitinib for refractory or transplant‐associated HLH [26, 27, 28]. Ruxolitinib selectively suppresses pro‐inflammatory cytokines with less myelosuppression, and modified HLH‐2004 protocols incorporating ruxolitinib have shown promising outcomes in transplant‐associated HLH [26, 29].

This particular case is an example of how difficult the diagnosis and treatment can become in case of secondary HLH occurring in a liver transplantation patient. If a postliver transplantation immunocompromised patient develops unexpected multi‐lineage pancytopenia, fever, hepatopathy, and high serum ferritin levels, HLH should be included in the differential diagnosis along with conditions like cellular rejection, primary graft dysfunction, cytopenias caused by medications, septic changes, and posttransplant lymphoproliferative disorders. The systematic use of HLH‐2004 criteria together with the H‐score makes it possible to increase the accuracy of diagnosing HLH in such a patient. The use of cytotoxic treatment involving etoposide poses a significant myelosuppressing threat, while in this particular case the development of invasive mucormycosis as a rare and quite often fatal condition occurring on the background of immunosuppression shows the fragility of the immune balance in these patients. Immunomodulatory treatment with ruxolitinib could prove to be effective in the future. Earlier recognition, multidisciplinary management, and heightened vigilance for invasive fungal infections during HLH‐directed therapy are critical to improving outcomes.

## Author Contributions

**Saif Ali Malik:** conceptualization, data curation, formal analysis. **Muhammad Hassaan Javaid:** methodology, project administration. **Syed Sibt E Haider:** resources, software, supervision. **Hajirah Ghairat:** validation, visualization, writing – original draft. **AbuBakar Hafeez Bhatti:** visualization, writing – original draft, writing – review and editing. **Hasibullah Aminpoor:** visualization, writing – original draft, writing – review and editing.

## Funding

The authors have nothing to report.

## Ethics Statement

The treating institution's ethics committee determined that formal ethical approval was not required for a single case report.

## Consent

Written informed consent for publication was obtained from the patient's next of kin following her death, in accordance with institutional guidelines.

## Conflicts of Interest

The authors declare no conflicts of interest.

## Data Availability

The data supporting the findings of this case report are available from the corresponding author upon reasonable request.

## References

[1] J. Chesner , T. D. Schiano , M. I. Fiel , and J. F. Crismale , “Hemophagocytic Lymphohistiocytosis Occurring After Liver Transplantation: A Case Series and Review of the Literature,” Clinical Transplantation 35, no. 8 (2021): e14340. 34159642 10.1111/ctr.14392

[2] N. Iseda , T. Yoshizumi , T. Toshima , et al., “Hemophagocytic Syndrome After Living Donor Liver Transplantation: A Case Report With a Review of the Literature,” Surgical Case Reports 4, no. 1 (2018): 101. 30159641 10.1186/s40792-018-0505-5 PMC6115321

[3] G. Griffin , S. Shenoi , and G. C. Hughes , “Hemophagocytic Lymphohistiocytosis: An Update on Pathogenesis, Diagnosis, and Therapy,” Best Practice & Research. Clinical Rheumatology 34, no. 4 (2020): 101515. 32387063 10.1016/j.berh.2020.101515

[4] M. Ramos‐Casals , P. Brito‐Zerón , A. López‐Guillermo , M. A. Khamashta , and X. Bosch , “Adult Haemophagocytic Syndrome,” Lancet 383, no. 9927 (2014): 1503–1516. 24290661 10.1016/S0140-6736(13)61048-X

[5] M. B. Jordan , C. E. Allen , S. Weitzman , A. H. Filipovich , and K. L. McClain , “How I Treat Hemophagocytic Lymphohistiocytosis,” Blood 118, no. 15 (2011): 4041–4052. 21828139 10.1182/blood-2011-03-278127 PMC3204727

[6] L. Raffray , L. Couzi , J. F. Viallard , et al., “Mycophenolate Mofetil: A Possible Cause of Hemophagocytic Syndrome Following Renal Transplantation?,” American Journal of Transplantation 10, no. 10 (2010): 2378–2379. 20840482 10.1111/j.1600-6143.2010.03254.x

[7] S. Xu and K. He , “Hemophagocytic Lymphohistiocytosis After Solid Organ Transplantation: A Challenge for Clinicians,” Transplantation Immunology 83 (2024): 102007. 10.1016/j.trim.2024.102007 38307154

[8] A. Meki , D. O'Connor , C. Roberts , and J. Murray , “Hemophagocytic Lymphohistiocytosis in Chronic Lymphocytic Leukemia,” Journal of Clinical Oncology 29, no. 24 (2011): e685–e687. 21709200 10.1200/JCO.2011.35.6139

[9] M. M. Lo , J. Q. Mo , B. P. Dixon , and K. A. Czech , “Disseminated Histoplasmosis Associated With Hemophagocytic Lymphohistiocytosis in Kidney Transplant Recipients,” American Journal of Transplantation 10, no. 3 (2010): 687–691. 20121728 10.1111/j.1600-6143.2009.02969.x

[10] J. K. Preiksaitis , D. C. Brennan , J. Fishman , and U. Allen , “Canadian Society of Transplantation Consensus Workshop on Cytomegalovirus Management in Solid Organ Transplantation,” American Journal of Transplantation 5, no. 2 (2005): 218–227. 15643981 10.1111/j.1600-6143.2004.00692.x

[11] L. Å. Rolsdorph , K. A. Mosevoll , L. Helgeland , and H. Reikvam , “Concomitant Hemophagocytic Lymphohistiocytosis and Cytomegalovirus Disease: A Case‐Based Systematic Review,” Frontiers in Medicine 9 (2022): 819465. 35514747 10.3389/fmed.2022.819465 PMC9063453

[12] K. Chevalier , J. Schmidt , P. Coppo , L. Galicier , N. Noël , and O. Lambotte , “Hemophagocytic Lymphohistiocytosis Associated With Cytomegalovirus Infection: 5 Cases and a Systematic Review of the Literature,” Clinical Infectious Diseases 76, no. 2 (2023): 351–358. 35974465 10.1093/cid/ciac649

[13] A. Pedicelli , R. P. Michel , and N. Krassakopoulos , “Cytomegalovirus‐Induced Hemophagocytic Lymphohistiocytosis in an Immunocompromised Patient With Inflammatory Bowel Disease,” Case Reports in Hematology 2024 (2024): 6964818. 38596354 10.1155/2024/6964818 PMC11003789

[14] K. Lehmberg , K. E. Nichols , J. I. Henter , et al., “Consensus Recommendations for the Diagnosis and Management of Hemophagocytic Lymphohistiocytosis Associated With Malignancies,” Haematologica 100, no. 8 (2015): 997–1004. 26314082 10.3324/haematol.2015.123562 PMC5004414

[15] J. I. Henter , A. Horne , M. Aricó , et al., “HLH‐2004: Diagnostic and Therapeutic Guidelines for Hemophagocytic Lymphohistiocytosis,” Pediatric Blood & Cancer 48, no. 2 (2007): 124–131. 16937360 10.1002/pbc.21039

[16] V. S. Tammisetti , S. R. Prasad , N. Dasyam , C. O. Menias , and V. Katabathina , “Immunosuppressive Therapy in Solid Organ Transplantation: Primer for Radiologists and Potential Complications,” Radiologic Clinics of North America 61, no. 5 (2023): 913–932. 37495297 10.1016/j.rcl.2023.04.010

[17] O. Abughanimeh , A. Qasrawi , and M. Abu Ghanimeh , “Hemophagocytic Lymphohistiocytosis Complicating Systemic Sarcoidosis,” Cureus 10, no. 6 (2018): e2838. 30131931 10.7759/cureus.2838 PMC6101461

[18] L. Fardet , L. Galicier , O. Lambotte , et al., “Development and Validation of the HScore, a Score for the Diagnosis of Reactive Hemophagocytic Syndrome,” Arthritis & Rhematology 66, no. 9 (2014): 2613–2620. 10.1002/art.38690 24782338

[19] Y. Song , J. Qiao , G. Giovanni , et al., “Mucormycosis in Renal Transplant Recipients: Review of 174 Reported Cases,” BMC Infectious Diseases 17, no. 1 (2017): 283. 28420334 10.1186/s12879-017-2381-1 PMC5395857

[20] A. M. Schram and N. Berliner , “How I Treat Hemophagocytic Lymphohistiocytosis in the Adult Patient,” Blood 125, no. 19 (2015): 2908–2914. 25758828 10.1182/blood-2015-01-551622

[21] X. Luo , C. Zhou , C. Ji , et al., “Hypofibrinogenemia Is an Independent Predictor of Hemophagocytic Lymphohistiocytosis in Children With Sepsis,” Scientific Reports 13, no. 1 (2023): 17936. 37863910 10.1038/s41598-023-44628-z PMC10589207

[22] W. Jeong , C. Keighley , R. Wolfe , et al., “The Epidemiology and Clinical Manifestations of Mucormycosis: A Systematic Review and Meta‐Analysis of Case Reports,” Clinical Microbiology and Infection 25, no. 1 (2019): 26–34. 30036666 10.1016/j.cmi.2018.07.011

[23] D. Loubet , B. Sarton , L. Lelièvre , et al., “Fatal Mucormycosis and Aspergillosis Coinfection Associated With Haemophagocytic Lymphohistiocytosis: A Case Report and Literature Review,” Journal de Mycologie Médicale 33, no. 1 (2023): 101325. 36270214 10.1016/j.mycmed.2022.101325

[24] E. Palomba , M. Colaneri , C. Azzarà , et al., “Epidemiology, Clinical Manifestations, and Outcome of Mucormycosis in Solid Organ Transplant Recipients: A Systematic Review of Reported Cases,” Open Forum Infectious Diseases 11, no. 6 (2024): ofae043. 38887489 10.1093/ofid/ofae043 PMC11181195

[25] L. Jarchin , J. Chu , M. Januska , P. Merola , and R. Arnon , “Autoimmune Hemolytic Anemia: An Unusual Presentation of Hemophagocytic Lymphohistiocytosis in a Pediatric Post‐Liver Transplant Patient,” Pediatric Transplantation 22, no. 7 (2018): e13247. 10.1111/petr.13281 30129086

[26] K. He , S. Xu , L. Shen , X. Chen , Q. Xia , and Y. Qian , “Ruxolitinib as Adjunctive Therapy for Hemophagocytic Lymphohistiocytosis After Liver Transplantation: A Case Report and Literature Review,” Journal of Clinical Medicine 11, no. 21 (2022): 6308. 36362534 10.3390/jcm11216308 PMC9656798

[27] L. Scholz , F. Posch , E. Schulz , and M. Gornicec , “Ruxolitinib, IV Immunoglobulin, and High‐Dose Glucocorticoids for Critically Ill Adults With Secondary Hemophagocytic Lymphohistiocytosis: A Single‐Center Observational Pilot Study,” Critical Care Explorations 6, no. 2 (2024): e1046. 38511127 10.1097/CCE.0000000000001046 PMC10954049

[28] Y. Wu , X. Sun , K. Kang , et al., “Hemophagocytic Lymphohistiocytosis: Current Treatment Advances, Emerging Targeted Therapy and Underlying Mechanisms,” Journal of Hematology & Oncology 17 (2024): 106. 39511607 10.1186/s13045-024-01621-x PMC11542428

[29] C. Salvador , B. Meister , H. Larcher , R. Crazzolara , and G. Kropshofer , “Hemophagocytic Lymphohistiocytosis After Allogeneic Bone Marrow Transplantation During Chronic Norovirus Infection,” Hematological Oncology 32, no. 2 (2014): 102–106. 23922241 10.1002/hon.2052

